# Performance characteristics and costs of serological tests for brucellosis in a pastoralist community of northern Tanzania

**DOI:** 10.1038/s41598-021-82906-w

**Published:** 2021-03-09

**Authors:** AbdulHamid S. Lukambagire, Ângelo J. Mendes, Rebecca F. Bodenham, John A. McGiven, Nestory A. Mkenda, Coletha Mathew, Matthew P. Rubach, Philoteus Sakasaka, Davis D. Shayo, Venance P. Maro, Gabriel M. Shirima, Kate M. Thomas, Christopher J. Kasanga, Rudovick R. Kazwala, Jo E. B. Halliday, Blandina T. Mmbaga

**Affiliations:** 1 College of Veterinary Medicine and Biomedical Sciences, Sokoine University of Agriculture, Morogoro, Tanzania; 2 Institute of Biodiversity, Animal Health and Comparative Medicine, College of Medical Veterinary and Life Sciences, University of Glasgow, Glasgow, G12 8QQ UK; 3 OIE/FAO Brucellosis Reference Laboratory, Department of Bacteriology, Animal and Plant Health Agency, Surrey, UK; 4 Endulen Hospital, Ngorongoro, Tanzania; 5 Kilimanjaro Christian Medical Center, Moshi, Tanzania; 6 Duke Global Health Institute, Durham, NC USA; 7 Kilimanjaro Clinical Research Institute-Biotechnology Laboratory, Moshi, Tanzania; 8 Regional Health Management Team, Arusha, Tanzania; 9 Kilimanjaro Christian Medical University College, Moshi, Tanzania; 10 The Nelson Mandela African Institution for Science and Technology, Arusha, Tanzania; 11 Centre for International Health, Dunedin School of Medicine, University of Otago, Dunedin, New Zealand

**Keywords:** Bacteriology, Infectious-disease diagnostics, Policy and public health in microbiology, Laboratory techniques and procedures, Infection, Bacterial infection, Bacterial infection, Diagnosis, Health care economics, Health policy, Epidemiology, Population screening

## Abstract

The control of brucellosis across sub-Saharan Africa is hampered by the lack of standardized testing and the use of tests with poor performance. This study evaluated the performance and costs of serological assays for human brucellosis in a pastoralist community in northern Tanzania. Serum collected from 218 febrile hospital patients was used to evaluate the performance of seven index tests, selected based on international recommendation or current use. We evaluated the Rose Bengal test (RBT) using two protocols, four commercial agglutination tests and a competitive enzyme-linked immunosorbent assay (cELISA). The sensitivity, specificity, positive predictive value, negative predictive value, Youden’s index, diagnostic accuracy, and per-sample cost of each index test were estimated. The diagnostic accuracy estimates ranged from 95.9 to 97.7% for the RBT, 55.0 to 72.0% for the commercial plate tests, and 89.4% for the cELISA. The per-sample cost range was $0.69–$0.79 for the RBT, $1.03–$1.14 for the commercial plate tests, and $2.51 for the cELISA. The widely used commercial plate tests performed poorly and cost more than the RBT. These findings provide evidence for the public health value of discontinuing the use of commercial agglutination tests for human brucellosis in Tanzania.

## Introduction

Brucellosis is considered to be the most widespread bacterial zoonosis of both veterinary and public health importance globally^1^. Over 500,000 new human cases are reported annually worldwide, mostly in low- and middle-income countries (LMICs)^2^. This figure is suspected to be an underestimation of the true incidence of the disease^2, 3^. Brucellosis is caused by bacteria of the genus *Brucella* and, in humans, the disease is most commonly caused by *B. abortus*, *B. melitensis,* and *B. suis*^4^. Human brucellosis cases are characterized by acute febrile illness that can progress to a chronic disease characterized by flu-like symptoms and musculoskeletal pain^5^. The occurrence of clinical signs in humans is highly variable, but an estimated 78% of patients experience fever and around 50% experience chronic musculoskeletal symptoms^6^. Up to 5% of acute cases suffer severe life-threatening complications^1^.

Given the wide variety of clinical manifestations of human brucellosis, diagnosis cannot be made solely on clinical grounds^7–9^. The United States Centers for Disease Control and Prevention (CDC) case definition for a confirmed brucellosis case is a clinically compatible illness with definitive laboratory evidence^7^. Definitive laboratory evidence is defined as a positive culture and identification of *Brucella* spp. from clinical samples or a four-fold or greater rise in *Brucella* antibody titer between acute and convalescent-phase serum samples collected at least two weeks apart^6,9^. In addition, probable cases are defined by the CDC as a clinically compatible illness with at least one of the following: epidemiologically linked to a confirmed *Brucella* case or having presumptive laboratory evidence. Presumptive laboratory evidence is defined as an antibody titer of ≥ 1:160 by serum agglutination test (SAT) or *Brucella* micro-agglutination test in one or more serum specimens obtained after onset of symptoms^9^. There are multiple case definitions in guidelines published by international organizations, in national surveillance programs, or in the scientific literature^10–15^. None of these case definitions allows the identification of all true brucellosis cases because of the imperfect performance of the recommended tests and variation in their performance at different stages of disease.

Healthcare facilities and laboratories in low-resource settings where brucellosis is endemic face several challenges in the diagnosis of human brucellosis. The recommended diagnostic methods (*e.g.* culture and serological testing of paired sera using the SAT) are technically demanding, have relatively slow turnaround times, are expensive, and are often not available in many endemic settings. Many commercially available plate agglutination tests, also known as rapid or febrile antigen *Brucella* agglutination tests, are widely used in human health facilities in the East Africa region, likely due to their perceived affordability and simplicity^16–19^. A small number of studies have shown that several commercial plate agglutination tests are inadequate for the diagnosis of brucellosis, but many different tests are currently available and in use across the region^16–18^. If the performance of all or many of the different tests of this type is as poor as the small number of existing studies suggest, the potential consequences of misdiagnosis are considerable. Thus, more evidence is needed to accurately quantify the performance characteristics of the range of kits in use and their affordability^16,20,21^. Although infections caused by *B. abortus* and *B. melitensis* cannot be differentiated with standard serological assays, *i.e.* those detecting antibodies to the smooth lipopolysaccharide (sLPS) of *Brucella* spp., some commercially available kits include separate *abortus* and *melitensis* suspensions, incorrectly suggesting that they can be used for this purpose^22,23^.

The Rose Bengal plate test (RBT), used in conjunction with confirmatory assays, such as culture or additional serological tests, is recommended by multiple international organizations for the diagnosis of human brucellosis^6,7,9^. There is considerable existing literature confirming the value of RBT in multiple contexts^19,24–29^. False negativity due to prozones (partial or no agglutination at low serum dilutions and complete agglutination at higher serum dilutions) and inability to detect non-agglutinating antibodies (IgA/IgG) have been raised as concerns with the RBT^20,30^. However, previous studies have found limited impact of prozones^31,32^. Although the RBT has been shown to be highly specific, there are recognized challenges for its use in clinical settings. False positivity can occur due to cross-reactivity with non-target pathogens or due to detection of antibodies attributable to previous exposure rather than being related to current illness, which is a significant challenge in brucellosis-endemic areas^31^. Other serological tests that employ the O-polysaccharide antigen (*e.g.* complement fixation test, SAT, and some ELISA kits) share some of these challenges. Application of the RBT with serial dilutions and use of a 1:8 titer cut-off (RBT 1:8) as opposed to reading the result using an undiluted sample (RBT 1:2) has been shown to improve the specificity of the test in healthy contacts of cases, without any significant decrease in sensitivity or increase in complexity, time for completion or cost^31^.

Competitive format ELISA tests have previously shown high sensitivity (98.3%) and specificity (99.7%) for detecting human brucellosis^33^ and have the advantage of allowing the use of the same assay to detect antibodies in various livestock hosts^34^ as well as in humans^33,35,36^. In comparison to rapid format tests, ELISA tests often show good performance, but the higher cost and infrastructural requirements mean they are often used as a second-line test in low-resource settings^37,38^. Several previous studies in Tanzania and the broader region have used the competitive ELISA (cELISA) evaluated in this study in combination with the RBT to test for brucellosis in humans, particularly in pastoralist communities^39–42^. The cELISA evaluated in this study has no recommended reference cut-off for use in human testing, having been developed and validated for livestock testing. The use of this specific kit for human testing therefore requires evaluation of the cut-off in different human populations and contexts.

Several measures can be used to characterize the diagnostic performance of serological tests, namely the sensitivity and specificity, positive and negative predictive values, diagnostic accuracy (the percentage of cases correctly diagnosed), Receiver Operating Characteristic (ROC) curves, and the Youden’s Index^43^. Different measures relate to the different aspects of the diagnostic process, such as the ability to correctly discriminate between samples from diseased and non-diseased individuals (sensitivity, specificity, Youden’s index (YI), area under the curve), and predictive ability (diagnostic accuracy, predictive values)^43,44^. The positive and negative predictive values and the diagnostic accuracy of a test all vary according to the prevalence of disease^43,45^. This variation by prevalence limits estimates of these metrics to the specific population evaluated and complicates extrapolations to other populations. While the sensitivity, specificity, YI, and area under the curve do not vary by prevalence and can be extrapolated to other populations, estimates of these metrics are also affected by population-specific disease dynamics, limiting inference between populations^43,44^. Diagnostic accuracy is commonly used alongside cost estimates to assess the cost-effectiveness of diagnostic tests in a specific geographical area^46–48^. Interpretation of the test accuracy metric should always be weighed considering other measures of prevalence-independent diagnostic performance^45,48,49^.

The cost of brucellosis diagnosis is an important element of the total public and private impacts of the disease^6,40,43^. Repeat visits to health facilities due to recurring illness, coupled with repeat testing due to poor test accuracy, substantially add to the costs of diagnosis, which are borne by the patient in most LMICs^43,46^. The running costs of currently available test options in northern Tanzania have not been fully evaluated. A comprehensive evaluation of these costs is needed to improve the cost-effectiveness of brucellosis diagnosis in the region, which is essential to mitigate some of the impacts of the disease.

The national surveillance guidelines for brucellosis in Tanzania recommend that all patients presenting to health facilities with brucellosis-consistent symptoms should be tested with RBT followed by a confirmatory serological test^10,38,50^. However, the RBT is not widely used in health facilities. Instead, a range of other tests commercially available on the Tanzanian market is used^51,52^. This study aimed to evaluate the diagnostic performance characteristics and running costs of the tests that are currently in use for human brucellosis in northern Tanzania and the wider region. Here, we include four commercial plate agglutination tests, the recommended RBT, and a cELISA kit. The outcomes of this assessment are expected to inform policy for the diagnosis and management of human brucellosis in Tanzania and other similar settings.

## Methods

### Study design

This study estimated the diagnostic performance of seven assays (henceforth referred to as index tests) using a set of sera from a study conducted to determine the prevalence of brucellosis amongst patients presenting to hospital with febrile illness. Patients were considered brucellosis cases if they met the CDC’s case definition for either a probable or confirmed case^15^. The case population thus included cases defined by culture positivity, SAT seroconversion, or high SAT titre (of ≥ 1:160 in acute, convalescent or both serum samples). Acute samples from all participants were collected at the time of hospital presentation when all individuals had documented fever. Full details of the patient population are described elsewhere^15^ and full details of the diagnostic testing performed for all participants are given in the accompanying data file (see Data Availability section). All samples used in this study were derived from blood samples collected at presentation to hospital, prior to any clinical intervention. All the tests were performed at the Kilimanjaro Clinical Research Institute—Biotechnology Laboratory in Moshi, Tanzania.

### Study population

Febrile patients presenting at the Endulen Hospital in the Ngorongoro Conservation Area between August 2016 and November 2017 were eligible to enroll in the previous prevalence study^15^. Inclusion criteria were: (1) age of two years or older and (2) reported fever within the past 72 h or a tympanic temperature of ≥ 38 °C at presentation. In total, 14 (6.1%) of 230 consecutively enrolled participants met the study definition for a probable or confirmed brucellosis case. Full details of the patient population, enrolment processes, patient testing and treatment are given elsewhere^15^.

### Data collection and tests evaluated

Out of 230 previously collected acute-phase serum samples, 218 had sufficient volume for completion of all evaluated tests and were included in this study. All samples excluded due to insufficient volume were collected from participants classified as negative for brucellosis case status^15^. In the population of 218 individuals evaluated for this study, (1) culture was performed in 186, eight (4.3%) of which had a positive result, and (2) SAT was performed in all (in both acute and convalescent-phase sera), twelve (6.4%) of which were positive (with a SAT titer ≥ 1:160). Of these twelve, one patient (0.5%) sero-converted (four-fold or greater rise in titer)^15^. Two cases that did not meet the SAT criteria in the case definition (either a SAT titer ≥ 1:160 or a four-fold or greater rise) were identified through culture. Out of the ten patients that met the SAT criteria in the case definition and had culture performed, six had a positive culture result.

The index tests for this study were performed by individuals who were blinded to the results of the previous testing and patient clinical information. The index tests evaluated were the standard RBT protocol (RBT 1:2)^31^, the RBT modified protocol with a 1/4 serum pre-dilution (RBT 1:8)^31^, four commercial plate agglutination tests available on the local market in Tanzania, and a cELISA kit used previously for human brucellosis testing studies in the region. For RBT 1:2, the test was performed following standard guidelines, testing serum samples with an equal volume of antigen^31^ (Rose Bengal antigen, RA 0060, Animal and Plant Health Agency (APHA)-Scientific, Weybridge-UK). For all samples classified as positive with RBT 1:2, doubling dilutions of serum (in buffered saline) were made from neat (1/1, reported as RBT 1:2) to 1/128 and each dilution tested with an equal volume (30 μL) of the Rose Bengal antigen. Diluted sera and antigen were mixed with a sterile wooden toothpick and gently rocked at room temperature for eight minutes. Any sample with visible agglutination observed at a titer of 1:8 was considered positive by the modified RBT 1:8 test. Positive and negative controls (APHA RAB1003—*Brucella abortus* positive control serum and RAB0701—*Brucella abortus* negative control serum) were run in parallel with all RBT test batches.

The manufacturer’s details of the four commercial plate agglutination tests evaluated were as follows: Amitech (Amitech Diagnostics, Ontario-Canada); Arkray (Arkray Healthcare Pvt., Surat-India); Eurocell (Euromedi Equip, Middlesex-UK); and, Fortress (Fortress Diagnostics, Antrim-UK). The four commercial plate agglutination tests were run as per kit instructions for the rapid, qualitative (screening) and semi-quantitative slide assays. The plate agglutination test protocols were identical except for the volumes of serum and antigen used. In all cases, equal volumes of serum and antigen were mixed. When a kit contained more than one antigen, sera were tested with each antigen included (Amitech *B. abortus* antigen at 50 μL serum and antigen volumes; Arkray stained *B. abortus* [15SA402-05] and *B. melitensis* [15SA403-05] suspension at 20 μL serum and antigen volumes; Eurocell *B. abortus* antigen at 50 μL serum and antigen volumes; Fortress Febrile *B. abortus* and *B. melitensis* [FEBAMP05] at 80 μL serum and antigen volumes). All kit protocols refer to controls, but only the Arkray and Fortress kits included controls when purchased from local suppliers. For this study, the positive controls provided with the Arkray and Fortress kits, the APHA *B. abortus* positive control serum used in the RBT tests, and a negative control were run on every test plate (*i.e.* four common controls in all test runs). For each combination of serum and antigen, equal volumes were mixed on a clean, white tile and rocked for one minute (except for the Fortress test, which was read after two minutes) at room temperature as per kit instructions. For each antigen, sera showing agglutination were further subjected to semi-quantitative titer testing with that same antigen as per kit instructions. Briefly, 80, 40, 20, 10, and five μL of serum were mixed with one drop of antigen (using the kit-provided dropper in each case) on a clean, white tile. Each reaction was rocked for one minute (except for the Fortress test, which was read after two minutes) at room temperature. Although samples were tested with each antigen included in the kits, data were analyzed in terms of *Brucella* spp. antibody detection only. A sample was classified as positive for *Brucella* spp. antibody detection by a given commercial plate agglutination test kit if agglutination was observed with a serum volume of 20 μL or less, as per kit instructions.

The cELISA kit evaluated was the COMPELISA400 that uses *B. melitensis* 16 M sLPS antigen (APHA Scientific), which was run as per kit instructions, as described elsewhere^53^. The optical density (OD) was read on an automated ELISA microplate reader (MultiSkan FC, Thermo Scientific, Germany) at 450 nm wavelength. A Receiver Operating Characteristic (ROC) curve analysis was carried out as described elsewhere^33,45,54^ using the R package ‘ROCit’^55^ to determine a suitable cut-off of the cELISA as applied to this human population. A two-graphs ROC was also produced (S1). A sample was considered cELISA positive for anti-*Brucella* antibodies if the sample OD was less than or equal to the optimal percentage of the OD of the four conjugate control wells (the cut-off), as estimated by the ROC analysis.

### Data analysis

All test results were compiled in Microsoft Excel. All data analyses were performed using R statistical software 3.6.1^56^. Given the pre-defined brucellosis case status for each sample, the results for each of the seven index tests were classified as one of the following: true positive (TP), false positive (FP), true negative (TN), and false negative (FN).

Several measures of diagnostic test performance were calculated for each of the index tests. These measures, recorded in percentage values (except for the Youden’s Index, which is expressed between 0 and 1), were calculated as follows:

- Sensitivity = $$\frac{{{\text{TP}}}}{{{\text{TP}} + {\text{FN}}}}$$;
- Specificity = $$\frac{{{\text{TN}}}}{{{\text{TN}} + {\text{FP}}}}$$;
- Positive predicted value (PPV) = $\frac{{{\text{TP}}}}{{{\text{TP}} + {\text{FP}}}}$;
- Negative predicted value (NPV) = $\frac{{{\text{TN}}}}{{{\text{TN}} + {\text{FN}}}}$;
- Youden’s Index (YI) = sensitivity + specificity − 1;
- Diagnostic accuracy = $$\frac{{{\text{TP }} + {\text{TN}}}}{{{\text{TP}} + {\text{ TN}} + {\text{FP}} + {\text{FN}}}}$$.

For each measure, except the YI, 95% confidence intervals were computed using the exact method for binomial distributions^57^. For the YI, 95% confidence intervals were calculated using the R package ‘ThresholdROC’^58^. The exact binomial test for differences in the sensitivity or specificity of pairwise combinations of index tests was performed with the R package ‘DTComPair’^59^.

The cost of running each of the index tests was calculated using a tool developed by the WHO^60^. To calculate the cost of each test, we assumed that each test was run independently for all samples and the same general conditions for test usage (*e.g.* number of batches run per week, number of samples tested per batch). The costs were estimated for the following:—reagents and consumables;—equipment;—personnel;—facilities; and,—quality control. The estimates and sources of the prices used for these calculations are given in the supplementary material (S2). The key assumptions made for the calculation of the cost per sample for each test were based on the premise that testing would be performed in a clinical setting, with rapid feedback of results required and thus small sample numbers per testing batch. These assumptions were as follows:—time to run one testing batch of 60 min, except for RBT 1:2 (30 min), RBT 1:8 (35 min) and cELISA (120 min);—laboratory working hours per day (eight);—laboratory working days per year (312);—laboratory working weeks per year (52);—testing schedule (number of batches tested per week; six);—number of samples per batch (five); and, percentage of samples retested (10). For cELISA, additional estimates of cost per sample were calculated assuming 30 samples per batch (one batch per week) and 60 samples per batch (one batch per two weeks). Given the influence of these key assumptions upon outcome values, a probabilistic sensitivity analysis with 1000 iterations was carried out to assess the level of variability in the outcome measures with variation in these assumptions. The distributions and values explored in this analysis are reported in the supplementary material (S3).

## Research clearance and ethics

Approval to conduct the study was granted by the Tanzania Commission for Science and Technology, Tanzania Wildlife Research Institute and the Ngorongoro Conservation Area Authority. Ethical approval was granted by the Kilimanjaro Christian Medical Centre (KCMC) Ethics Committee (698), National Institute of Medical Research (NIMR), Tanzania (NIMR/HQ/R.8c/Vol. I/1140), University of Otago Human Ethics Committee (H17/052), and University of Glasgow College of Medical, Veterinary and Life Sciences Ethics Committee (200140149). The research was performed in accordance with the guidelines and regulations prescribed by the above organizations. Written informed consent for study participation was obtained from each participant and/or their legal guardian, using forms translated into Swahili and verbal translation into Maa when needed. All procedures were conducted according to recommended international standards and following manufacturer’s instructions.

## Results

### Participants

Of the 218 individuals included in this study, 93 (42.7%) were males. The age of participants ranged from 2 to 78 years, with a median of 27 years. Among 183 study participants for whom there were clinical diagnosis records, 76 (40.4%) presented with respiratory symptoms. Brucellosis was included in the initial diagnosis made at presentation for 36 (19.6%) of these 183 participants.

### Diagnostic performance characteristics

**Figure 1:**
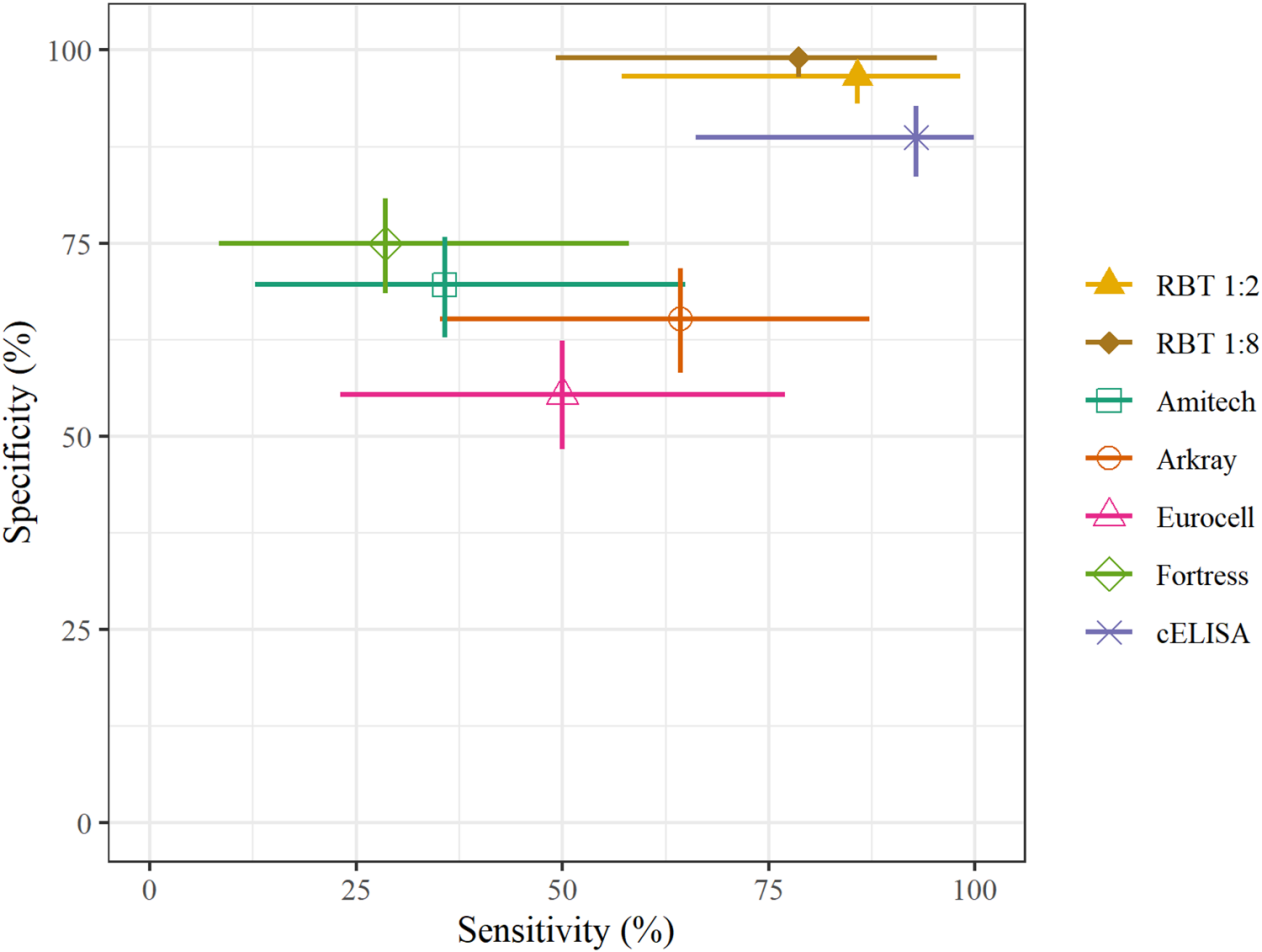
Point estimates of the sensitivity and specificity with 95% confidence intervals (horizontal and vertical lines) for each index test (n = 218). RBT: the Rose Bengal test. cELISA: competitive enzyme-linked immunosorbent assay.

The cross tabulation for each index test compared with the previously defined brucellosis case status of each individual is presented in Table 1. The optimal OD cut-off point for this population estimated using the ROC curve analysis was 56% of the OD of the conjugate control wells (S1), and this 56% cut-off was used to define sample cELISA results for this study. The results for all samples included in the study with all index tests and also additional RBT dilutions are given in the accompanying data file (see Data Availability section). The RBT 1:2 and RBT 1:8 had diagnostic accuracy estimates of 95.9% and 97.7%, respectively (Table 2). The four plate agglutination tests had diagnostic accuracy estimates ranging from 55.0 to 72.0%. The estimated accuracy of the cELISA was 89.4%. The estimated sensitivity and specificity for each index test are shown in Fig. 1. The sensitivity, specificity, PPV, NPV, YI, and diagnostic accuracy estimates for each index test are given in Table 2. The statistical significance of differences between the estimated sensitivity and specificity of each assay pair is shown in supplementary material (S4). According to this statistical analysis, the RBT 1:2, RBT 1:8, and cELISA had higher specificity than the four commercial agglutination tests. The RBT 1:2 had higher sensitivity than two (Amitech and Fortress) of the commercial agglutination tests. The cELISA had higher sensitivity than three of the commercial agglutination tests but not the Arkray test. The index and reference test results for each sample are shown in supplementary material (S5).

**Table 1:** Cross tabulation of the index test results by patient brucellosis case status (n = 218, of which 14 were brucellosis cases and 204 were non-brucellosis cases).

Index test	Index test result	Brucellosis case status			
		Non-brucellosis case	Brucellosis case	Total	Percentage positive by test (95% CI)
RBT 1:2	Negative	197	2	199	8.7 (5.3–13.3)
	Positive	7	12	19	
RBT 1:8	Negative	202	3	205	6.0 (3.2–10.0)
	Positive	2	11	13	
Amitech	Negative	142	9	151	30.7 (24.7–37.3)
	Positive	62	5	67	
Arkray	Negative	133	5	138	36.7 (30.3–43.5)
	Positive	71	9	80	
Eurocell	Negative	113	7	120	45.0 (38.2–51.8)
	Positive	91	7	98	
Fortress	Negative	153	10	163	25.2 (19.6–31.5)
	Positive	51	4	55	
cELISA	Negative	182	1	183	16.1 (11.4–21.6)
	Positive	22	13	35	

*RBT* the Rose Bengal test, *cELISA* competitive enzyme-linked immunosorbent assay, *CI* confidence interval.

**Table 2:** Performance characteristic estimates for each index test.

Test	Sensitivity % (95% CI)	Specificity % (95% CI)	PPV % (95% CI)	NPV % (95% CI)	YI (95% CI)	Accuracy % (95% CI)
RBT 1:2	85.7 (57.2–98.2)	96.6 (93.1–98.6)	63.2 (38.4–83.7)	99.0 (96.4–99.9)	0.82 (0.81–0.84)	95.9 (92.3–98.1)
RBT 1:8	78.6 (49.2–95.3)	99.0 (96.5–99.9)	84.6 (54.6–98.1)	98.5 (95.8–99.7)	0.78 (0.75–0.80)	97.7 (94.7–99.3)
Amitech	35.7 (12.8–64.9)	69.6 (62.8–75.8)	7.5 (2.5–16.6)	94.0 (89.0–97.2)	0.05 (0.02–0.09)	67.4 (60.8–73.6)
Arkray	64.3 (35.1–87.2)	65.2 (58.2–71.7)	11.3 (5.3–20.3)	96.4 (91.7–98.8)	0.29 (0.26–0.33)	65.1 (58.4–71.4)
Eurocell	50.0 (23.0–77.0)	55.4 (48.3–62.3)	7.1 (2.9–14.2)	94.2 (88.4–97.6)	0.05 (0.02–0.09)	55.0 (48.2–61.8)
Fortress	28.6 (8.4–58.1)	75.0 (68.5–80.8)	7.3 (2.0–17.6)	93.9 (89.0–97.0)	0.04 (0.01–0.07)	72.0 (65.6–77.9)
cELISA	92.9 (66.1–99.8)	89.2 (84.1–93.1)	37.1 (21.5–55.1)	99.5 (97.0–100.0)	0.82 (0.81–0.83)	89.4 (84.6–93.2)

*PPV* positive predictive value, *NPV* negative predictive value, *YI* Youden’s index reported to two decimal places, *RBT* the Rose Bengal test, *cELISA* competitive enzyme-linked immunosorbent assay, *CI* confidence interval.

### Diagnostic test costs

**Figure 2:**
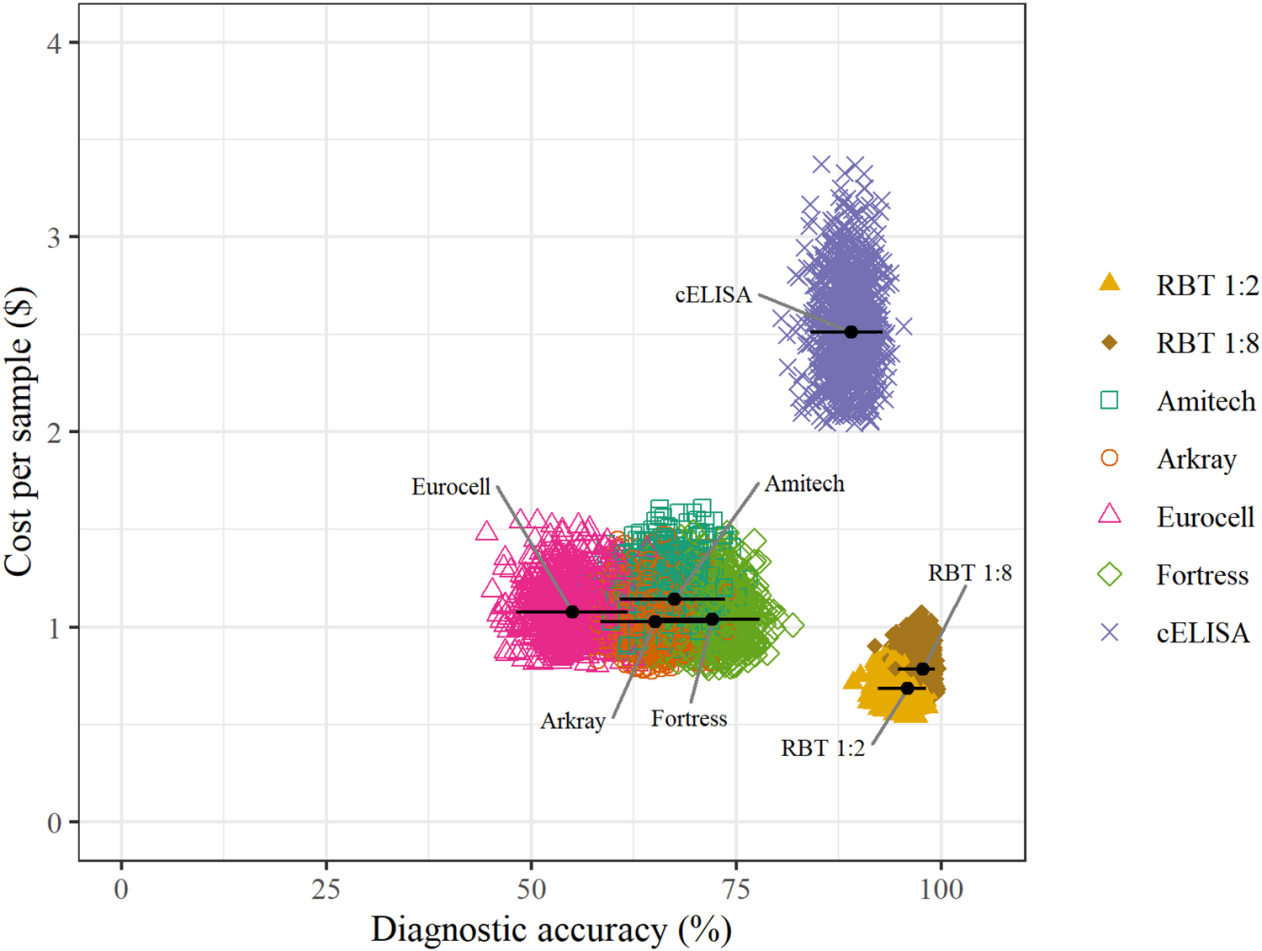
Probabilistic sensitivity analysis of the cost per sample of each of the index tests (estimates indicated by colored symbols) with their corresponding diagnostic accuracy estimate (black dots; 95% confidence intervals represented with black lines). *RBT* the Rose Bengal test, *cELISA* competitive enzyme-linked immunosorbent assay.

The estimated cost per sample of the seven index test options ranged from $0.69 for RBT 1:2 to $2.51 for cELISA (Table 3). The greatest proportion of component costs were made up by consumables and personnel. The higher cost per sample of the cELISA reflects longer test runtimes, requirement for specialized equipment, and higher cost per kit. All the plate agglutination assays were cheaper relative to the cELISA, with cost variation largely dependent on kit-specific consumables. Figure 2 shows the relationship between test diagnostic accuracy and cost per sample for the seven index test options evaluated. The probabilistic sensitivity analysis showed that plausible variation in the estimates of component costs did not affect the overall conclusions about the relative costs of these tests, based on their use as frontline options and the corresponding costing assumptions made. The RBT 1:2 and RBT 1:8 assays showed the highest accuracy and lowest cost (Fig. 2).

**Table 3:** Cost per sample in United States dollars and diagnostic accuracy estimates for each index test evaluated (with 95% confidence intervals; 95% CI).

Test	Cost per sample ($)						Accuracy % (95% CI)
	Consumables	Equipment	Personnel	Facilities	Quality control	Total	
RBT 1:2	0.34	< 0.001	0.19	0.07	0.09	0.69	95.9 (92.3–98.1)
RBT 1:8	0.39	< 0.001	0.22	0.09	0.09	0.79	97.7 (94.7–99.3)
Amitech	0.53	0.001	0.38	0.15	0.09	1.14	67.4 (60.8–73.6)
Arkray	0.41	0.001	0.38	0.15	0.09	1.03	65.1 (58.4–71.4)
Eurocell	0.46	0.001	0.38	0.15	0.09	1.08	55.0 (48.2–61.8)
Fortress	0.42	0.001	0.38	0.15	0.09	1.04	72.0 (65.6–77.9)
cELISA	1.36	0.003	0.76	0.30	0.09	2.51	89.4 (84.6–93.2)
cELISA^30^	1.09	0.001	0.13	0.05	0.09	1.36	“
cELISA^60^	1.07	< 0.001	0.06	0.02	0.09	1.24	“

The first seven rows indicate costs assuming testing of five samples per batch, six batches per week, and 52 weeks per year. Rows for cELISA^30^ and cELISA^60^ represent costs assuming testing of 30 samples per batch, one batch per week and 60 samples per batch, one batch per two weeks, for cELISA, respectively. CI: confidence interval. RBT: the Rose Bengal test. cELISA: competitive enzyme-linked immunosorbent assay.

## Discussion

Our data show that all the rapid commercial plate assays evaluated had poor diagnostic accuracy. In comparison, the RBT 1:2 and RBT 1:8 assays both had high diagnostic accuracy and also had lower costs per sample when applied to diagnose brucellosis in this population of Tanzanian pastoralists. The cELISA had high diagnostic accuracy but a higher cost per sample when evaluated as a frontline test. This study provides a strong rationale for replacing the rapid commercial plate assays with the RBT for frontline brucellosis testing in Tanzanian health facilities.

Our findings in this Tanzanian pastoralist population corroborate the results of earlier studies carried out elsewhere, where excellent diagnostic performance of RBT 1:2 (high sensitivity and specificity estimates within the 85–100% interval) was reported^24,27,29,61–64^. RBT specificity estimates may be underestimated in contexts where a positive test can occur due to previous exposure to *Brucella* spp., rather than active infection, or an active infection caused by a cross-reacting pathogen (e.g. *Y. enterocolitica* O:9, *Vibrio cholerae O:1, Francisella tularensis* or *Escherichia coli* O157)^20^. Compared to our reference case standard (including SAT and blood culture results), RBT 1:2 and RBT 1:8 both displayed high specificity. With RBT 1:8, the point estimate for specificity increased from 96.6 to 99.0%. However, the 95% confidence intervals on these estimates overlap. Five out of the seven false positive test results observed with RBT 1:2 were classified as true negatives with RBT 1:8. This leads to an increase of 21.4% in the PPV (from 63.2% for RBT 1:2 to 84.6% for RBT 1:8) (Table 2). The precision of these estimates is limited by the relatively small sample size available for this study, but a true difference between the two RBT protocols is likely to be important in clinical practice. Particularly in contexts where access to confirmatory tests is limited, a high PPV is a crucial attribute of a frontline test. The PPV determines the confidence with which health practitioners start patients on targeted treatments. For brucellosis, high PPV is particularly important, given the long duration of recommended treatment regimens, adverse effects of these regimens for patients, frequent involvement of restricted drugs, and frequent treatment failures^5,65,66^. A full evaluation of the cut-off used for the RBT was not performed as part of this study, in part due to the small proportion of positive individuals and thus limited data to robustly compare results at different dilutions. However, the data for all RBT results at serial dilution are shown in the accompanying data file (see Data Availability section). Further evaluation of the field performance of the RBT with different dilution cut-offs at scale could resolve this query. Future studies could also aim to inform selection of a preferred testing protocol for this context and shed light on the impacts of current misdiagnosis.

Our results showed that the widely used commercial plate agglutination tests have significantly lower specificity and diagnostic accuracy as compared to the RBT protocols. These findings agree with the small number of published evaluations of similar tests^16–18^. We estimate that the PPV of each of the commercial plate agglutination tests is at least six times lower than that of the RBT 1:2 (63.2%) and RBT 1:8 (84.6%). Given the relatively small sample size and low brucellosis case prevalence in this sample set, the sensitivity estimates obtained in this study have wide confidence intervals. However, the point estimates for sensitivity indicate that between 28.6% (Fortress) and 64.3% (Arkray) of the pre-defined brucellosis cases were classified as positive by the commercially available plate agglutination tests. Estimating the performance of RBT 1:2 and RBT 1:8 using the commercial plate agglutination tests as reference further highlights the difference in performance between these tests: (1) if the Eurocell test (the rapid commercial plate assay with highest percentage of samples positive and lowest estimated accuracy) was used as the reference for true case status, the estimated accuracy of both RBT 1:2 and 1:8 would be 53.7% (95% CI 46.8–60.4); (2) if the Fortress test (the rapid commercial plate assay with lowest percentage of samples positive and highest estimated accuracy) was used as the reference for true case status instead, the accuracy of RBT 1:2 and RBT 1:8 would be 69.7% (95% CI 63.2–75.7) and 72.5% (95% CI 66.0–78.3), respectively. Given the considerable existing literature on the performance of the RBT (1:2 and 1:8), these accuracy estimates are not plausible. These data further illustrate that the results of the commercial plate agglutination tests cannot be regarded as accurate indicators of true brucellosis case status. The proportion of individuals testing positive by the four commercial plate agglutination tests (Table 1) are implausibly high, when evaluated alongside the other tests and the existing literature on the brucellosis prevalence expected in this and other comparable populations^15,51,67^. These estimates are unlikely to be explained by previous exposure in this population^68–71^, and are more likely due to the low specificity of these tests. The higher sensitivity of RBT protocols (as compared to these commercial plate agglutination tests) is likely to be explained, at least partially, by the standardization of the antigen to OIE specification and the acid buffer used to suspend Rose Bengal stained *Brucella* cells. The acid buffering improves the ability of RBT to detect agglutinating and non-agglutinating antibodies irrespective of the stage of disease evolution^30^. Information on the pH of the buffers used with the commercially available plate agglutination tests is not included in the test kits. Our data provide further rationale for replacement of the poorly performing plate agglutination tests that are currently used in Tanzanian health facilities with RBT (RBT 1:2 or RBT 1:8), as recommended in national and international guidelines^6,7,9^.

Using the estimated optimal cut-off for human testing, the cELISA evaluated in this study was highly sensitive and specific in this population. The kit recommended cut-off for this cELISA, which has been applied for human testing previously^39–41^ uses a cut-off value of 60% of the OD obtained with conjugate control wells. This threshold value was originally optimized for livestock testing, and its application to human samples requires formal evaluation^33,34,72–74^. The estimated cut-off point based on the assay readings for this population and the pre-defined brucellosis case status (56% of the conjugate blank OD) fell close to the kit recommended value (60%). The high estimates of sensitivity and specificity generated from a small sample set provide a strong justification for a full validation of the cELISA, specifically including cut-off evaluation in a larger dataset that ideally also includes well-characterized patient samples known to span the different clinical stages of presentation of human brucellosis.

There are no publicly available data on the per-sample running costs of the RBT or alternative test options in northern Tanzania^75^. The cost of a diagnostic test can negatively impact its utility^20,37,46^, especially in rural, low-resource settings^5,8,20^. Our data suggest that RBT 1:2 is the cheapest option for frontline use among the evaluated tests. The RBT 1:8 has marginally increased costs as compared to the RBT 1:2 due to the additional time and consumables required for serum dilution, but this cost difference is trivial (Fig. 2). In addition to the poor diagnostic performance of the commercially available plate agglutination tests, they also cost more per sample as compared to the RBT 1:2 or RBT 1:8 (Fig. 2). The cELISA costs more per sample than any of plate agglutination tests evaluated under the common assumptions specified. However, the costs per sample for the cELISA are substantially reduced when samples are batched for testing (Table 3). The application of the cELISA, with batching of samples, is more likely to occur when used as a frontline test in larger health facilities. In this study, our primary aim was to assess the suitability of available options specifically for frontline use in a clinical setting, hence, assuming a small number of samples per batch. Under these circumstances, RBT 1:2 and RBT 1:8 were more affordable (and accurate) than any of the other evaluated test options.

The availability and use of a rapid, cheap, and accurate test for the diagnosis of human brucellosis are vital to minimize some of the impacts of brucellosis. The higher the test accuracy in particular, the lower the risk of delays in diagnosing true cases and, consequently, the lower the multiple downstream impacts of missed diagnoses. Among the population of individuals tested for brucellosis but who are not true cases, a higher test accuracy could also contribute to faster exclusion of brucellosis as a likely cause of illness. The large-scale deployment of a cheap and accurate test for brucellosis would also be key to strengthening surveillance capacity, therefore improving the quality of the data needed to plan, design, and deliver brucellosis control strategies. Our findings indicate that the RBT is a good candidate for national roll-out in Tanzania. Further evaluation of RBT implementation at scale is needed to assess, among other factors, reliability of the reagent supply chain, ability to ensure and maintain antigen quality in field conditions^76,77^ and overall test performance under field conditions. A regional or national scale evaluation could also provide evidence to inform the selection of the best candidate test for confirmatory testing in this context.

This study has several limitations. First, given the limited sample size and proportion of brucellosis cases in the population used for this study, the confidence intervals on many of the estimates of test sensitivity are wide and overlap in many cases. Second, we used serum of febrile patients from a pastoralist community, some of whom may have had previous exposure to *Brucella*^39,67,78^. We evaluated the performance of the index tests in this study with reference to sample status defined by SAT and culture tests that are estimated to have lower sensitivity than the RBT and some cELISA assays^20,31^. As a consequence, our estimates of the specificity and PPV of the index tests evaluated might be underestimated in comparison to their unobserved true performance in this population. Third, for the commercial plate agglutination tests, we used the semi-quantitative dilution protocols described in the test kit materials in all cases. In practice, these dilution protocols are rarely applied in health facilities, and test results are performed with neat serum testing only^51,52^. For this reason, our data may well over-estimate the specificity of the commercial plate agglutination tests as compared to their common use in practice. Finally, all of the diagnostic test data presented were generated in a research laboratory, and we have not evaluated the field performance of these tests.

## Conclusions

This evaluation of the diagnostic performance characteristics of tests for human brucellosis provides robust estimates of the markedly poor diagnostic performance of the commercial plate agglutination tests currently available and widely used in Tanzania. Our results suggest that data generated based on these currently used tests are likely to be highly inaccurate and that the systematic use of RBT (either RBT 1:2 or RBT 1:8) as the frontline test for human brucellosis in northern Tanzania would provide more accurate data on human brucellosis than is currently available. In addition, the per-sample costs of RBT 1:2 and RBT 1:8 were lower than any other test evaluated. Future studies to evaluate the feasibility and cost-effectiveness of national roll-out of RBT as the frontline brucellosis test in Tanzania are recommended. Standardized application of RBT for human brucellosis testing across Tanzania could have enormous value for both patient management and also for understanding the current distribution and burden of disease by improving disease surveillance data^10,50^.

## Supplementary Information

Supplementary Information.

## Data Availability

The datasets generated during and/or analysed during the current study are available in the Enlighten research data repository of the University of Glasgow (10.5525/gla.researchdata.1119).
