# Supplementary material for: Performance characteristics and costs of serological tests for brucellosis in a pastoralist community of northern Tanzania
Source: Sci Rep. 2021 Mar 9;11:5480. doi: 10.1038/s41598-021-82906-w (PMC7943594; doi:10.1038/s41598-021-82906-w)
Supplement: Supplementary file 1 — Supplementary Information. [file 41598_2021_82906_MOESM1_ESM.docx]

**Full title:** Performance characteristics and costs of serological tests for brucellosis in a pastoralist community of northern Tanzania.

AbdulHamid S. Lukambagire^1*^, Ângelo J. Mendes^2^, Rebecca F. Bodenham^2^, John A. McGiven^3^, Nestory A. Mkenda^4^, Coletha Mathew^1^, Matthew P. Rubach^5, 6^, Philoteus Sakasaka^6, 7^, Davis D. Shayo^8^, Venance P. Maro^5,9^, Gabriel M. Shirima^10^, Kate M. Thomas^7,11^, Christopher J. Kasanga^1^, Rudovick R. Kazwala^1^, Jo E.B. Halliday^2^, Blandina T. Mmbaga^5,6,7,9^.

*Correspondence to: [lukhamid@gmail.com](mailto:lukhamid@gmail.com)

1. College of Veterinary Medicine and Biomedical Sciences, Sokoine University of Agriculture, Morogoro, Tanzania
2. Institute of Biodiversity, Animal Health and Comparative Medicine, College of Medical Veterinary and Life Sciences, University of Glasgow, Glasgow G12 8QQ, United Kingdom
3. OIE/FAO Brucellosis Reference Laboratory, Department of Bacteriology, Animal & Plant Health Agency, Surrey, UK
4. Endulen Hospital, Ngorongoro, Tanzania
5. Kilimanjaro Christian Medical Center, Moshi, Tanzania
6. Duke Global Health Institute Durham, North Carolina, USA
7. Kilimanjaro Clinical Research Institute-Biotechnology Laboratory, Moshi, Tanzania
8. Regional Health Management Team, Arusha, Tanzania
9. Kilimanjaro Christian Medical University College, Moshi, Tanzania
10. The Nelson Mandela African Institution for Science and Technology, Arusha, Tanzania
11. Centre for International Health, Dunedin School of Medicine, University of Otago, Dunedin, New Zealand.

**Supplementary materials**


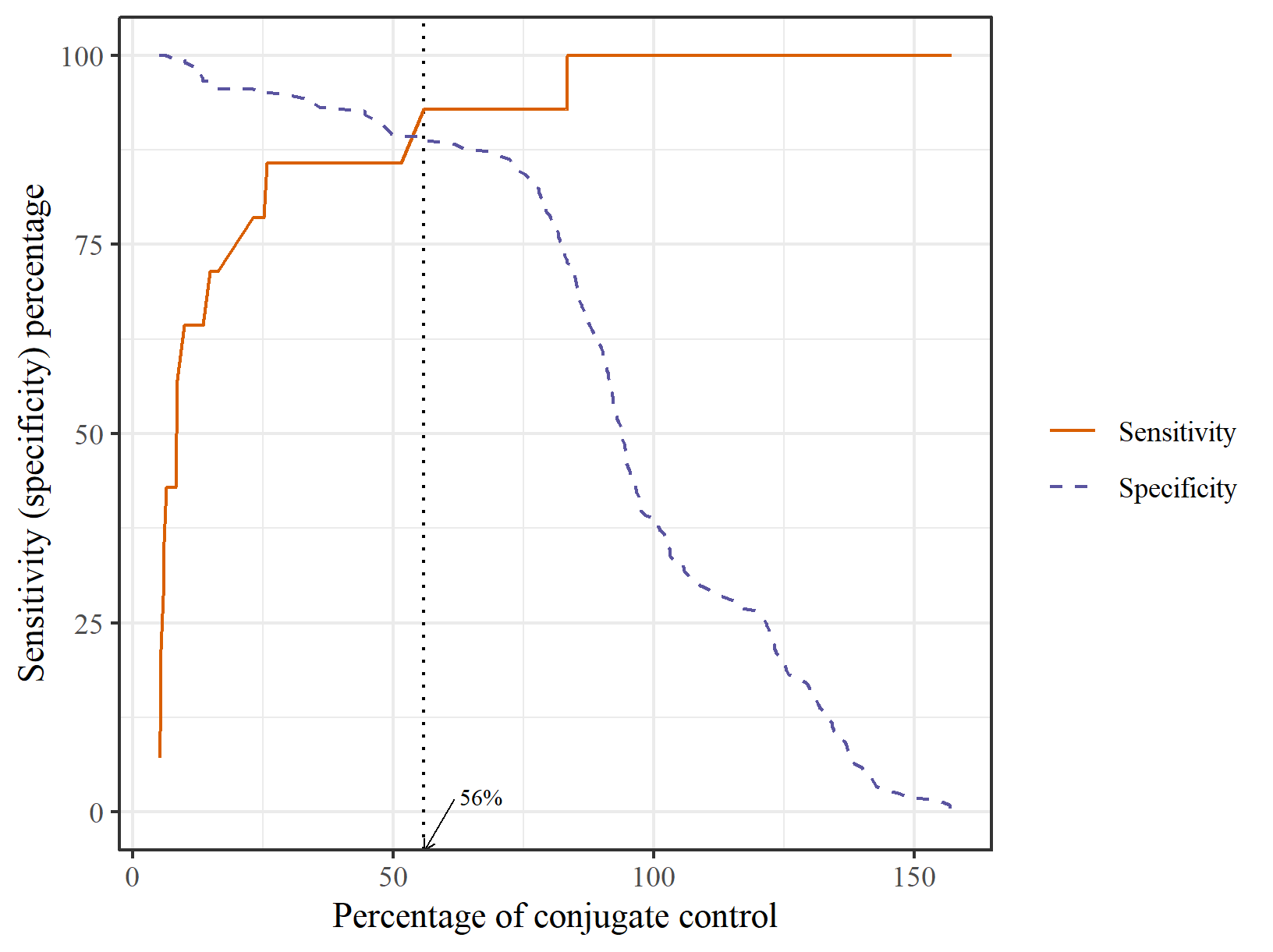


**S1. Two-Graphs Receiving Operating Characteristics.** The black dotted line indicates the threshold used as cut-off for classification. At this value, the sum of sensitivity and specific is maximized.

**S2. Pricing sources and details assumed for all component costs in cost estimation (currency: USD):** Price assumes purchase in Tanzania Shillings (conversion rate to 1 $ (USD) on 11/07/2019 from Central Bank of Tanzania at Spot Buying: 2,277.69 TZS), except for items purchased from APHA Scientific as the currency was the pound sterling (conversion rate to 1 £ (GBP) on 11/07/2019 from Central Bank of Tanzania at Spot Buying: 2,851.21 TZS); U/PS: units used per patient sample; U/QC: number of units for QC per batch (run); T/K: tests per kit; YL: 10 years of lifespan; MC/Y: maintenance cost per year in USD; PU/D: percentage use for method per day; RBT: the Rose Bengal test (both RBT 1:2 and RBT 1:8 protocols); cELISA: competitive enzyme-linked immunosorbent assay.

| **Cost category** | **Item** | **Reference / manufacturer** | **Price** | **Details** | | |
| --- | --- | --- | --- | --- | --- | --- |
| **Reagents and consumables** |  |  |  | **U/PS** | **U/QC** | **Other details** |
|  | Eurocell kit | 56/EME/1210/VER-01 | 7.03 | 1 | 2 | 100 T/K |
|  | Fortress kit | FEBAMP-FEBRILE v3 | 14.05 | 1 | 2 | 100 T/K; controls included |
|  | Arkray kit | 15SA402-05/3-05 | 13.17 | 1 | 2 | 100 T/K; controls included |
|  | Amitech kit | Amitech Diagnostics Inc. | 13.17 | 1 | 2 | 100 T/K |
|  | RBT antigen | APHA Scientific | 316.71 | 1 | 2 | 4,000 T/K |
|  | cELISA kit | APHA Scientific | 264.13 | 1 | 2 | 400 T/K; controls included |
|  | Eurocell controls | 56/EME/1210/VER-01 | 7.03 | 0 | 1 | 10 T/K |
|  | Amitech controls | Amitech Diagnostics Inc. | 5.71 | 0 | 1 | 10 T/K |
|  | RBT controls | APHA Scientific (RAB1003 and RAB 0701) | 65.09 | 0 | 1 | 4,000 T/K |
|  | Gloves | Fisher Scientific | 3.29 | 2 | 2 | pack of 100 units |
|  | Tips | ABS | 25.07 | 2 (except 4 for RBT 1:8 and 7 for cELISA) | 2 (except 7 for cELISA) | pack of 1,000 units of 1,000 μL without filter |
|  | Stirrer | Local | 0.22 | 1 (except 0 for cELISA) | 1 (except 0 for cELISA) | pack of 100 toothpicks |
|  | Saline | Local | 1.54 | 5 (except 2 for RBT 1:8, and 0 for RBT 1:2, Fortress and cELISA) | 1 (except 0 for RBT 1:2, Fortress and cELISA) | 1L of clinical grade saline; 1 unit = 0.05 ml |
|  | Tile | Local | 1.01 | 0.001 | 0.001 | 1 unit = 1 x 0.5 ft tile |
|  | Disposable gown | Fischer Scientific | 6.59 | 0.1 | 0.1 | pack of 25 units |
|  | Surface disinfectant wipes | Local | 7.50 | 0.1 | 0.1 | pack of 200 units |
|  | Hand towel roll | Local | 7.50 | 0.2 | 0.2 | 6 rolls of 100 m; 1 unit = 1 m length |
|  | Permanent marker | Local | 6.59 | 0.01 | 0.01 | pack of 12 units |
|  | Paper A4 | Local | 4.39 | 1 | 1 | pack of 500 units |
| **Equipment** |  |  |  | **YL** | **MC/Y** | **PU/D** |
|  | Refrigerator | Westpoint | 285.38 | 10 | 30 | 5 |
|  | Thermometer | Invitrogen | 15.00 | 10 | 1 | 1 |
|  | Computer with printer | Dell | 439.04 | 10 | 50 | 2 |
|  | Pipette 20-200 | Fischer Scientific | 150.00 | 5 | 5 | 10 |
|  | Pipette 10-100 | Fischer Scientific | 150.00 | 5 | 5 | 0 (except 10 for cELISA) |
|  | Pipette 100-1000 | Fischer Scientific | 150.00 | 5 | 5 | 0 (except 10 for cELISA) |
|  | ELISA reader^5^ | BenQ | 79.03 | 15 | 15 | 30 |
| **Personnel** | Annual salary | n/a | 3,951.37 | plus 20% of administration costs | | |
| **Facility (price per year)** | Rent | n/a | 5,000.00 | n/a | | |
|  | Building maintenance | n/a | 250.00 | n/a | | |
|  | Gas and heating | n/a | 0.00 | n/a | | |
|  | Water | n/a | 110.00 | n/a | | |
|  | Electricity | n/a | 110.00 | n/a | | |
|  | Internet and telephone | n/a | 50.00 | n/a | | |
|  | Other costs | n/a | 50.00 | n/a | | |
| **Quality control** | Annual proficiency testing panel | n/a | 100.00 | n/a | | |
|  | Annual audit | n/a | 350.00 | With 10 methods being audited each year | | |

**S3: Values and distributions assumed for the probabilistic sensitivity analysis.** Average time required to run a batch of (ATRB) Eurocell, Fortress, Arkray and Amitech: 60 minutes; ATRB RBT(1:2): 30 minutes; ATRB RBT(1:8): 35 minutes; ATRB cELISA: 120 minutes; Laboratory average working hours per day, days per year, weeks per year, runs per week, and tests per year: 8, 312, 52, 6, 1560; RBT: the Rose Bengal test (both RBT 1:2 and RBT 1:8 protocols); cELISA: competitive enzyme-linked immunosorbent assay.

| **Parameter** | **Value / distribution** | **Units** |
| --- | --- | --- |
| Average time required to run a batch of (ATRB) | Uniform (50,70) | Minutes |
| ATRB RBT (1:2) | Uniform (25,35) | Minutes |
| ATRB RBT (1:8) | ATRB RBT (1:2) + 5 | Minutes |
| ATRB cELISA | Uniform (110,130) | Minutes |
| Laboratory average working hours per day | Uniform (6,10) | Hours |
| Laboratory average working days per week | Uniform (5,7) | Days |
| Laboratory average working weeks per year | Fixed (52) | Weeks |
| Testing schedule (number of runs per week) | Uniform (5,7) | N/A |
| Annual salary | Gamma (7902,2) | USD |
| Rent / year | Gamma (1250,0.25) | USD |
| Building maintenance / year | Gamma (62.5,0.25) | USD |
| Water / year | Gamma (110,1) | USD |
| Electricity / year | Gamma (110,1) | USD |
| Internet and telephone / year | Gamma (25,0.5) | USD |
| Other costs / year | Gamma (25,0.5) | USD |
| Annual proficiency testing panel | Gamma (50,0.5) | USD |
| Annual audit | Gamma (175,0.5) | Units |

**S4**. **Pairwise comparison of sensitivity (top / right; blue cells) and specificity (bottom / left; red cells) estimates from each combination of index tests (n=218).** All pairwise metrics were tested by differences between proportions. RBT - the Rose Bengal test (both RBT 1:2 and RBT 1:8 protocols); cELISA - competitive enzyme-linked immunosorbent assay. The statistical significance of the test of pairwise differences are shown as follows: ^*^p-value < 0.05; ^**^p-value < 0.01; ^***^p-value < 0.001.

|  |  | **Test** | | | | | | | | | | | | | |
| --- | --- | --- | --- | --- | --- | --- | --- | --- | --- | --- | --- | --- | --- | --- | --- |
|  |  | **RBT 1:2** | | **RBT 1:8** | | | **Amitech** | | **Arkray** | | | **Eurocell** | **Fortress** | **cELISA** | |
| **Test** | **RBT 1:2** |  | |  | | | * | |  | | |  | * |  | |
|  | **RBT 1:8** |  | |  | | |  | |  | | |  | * |  | |
|  | **Amitech** | *** | | *** | | |  | |  | | |  |  | ** | |
|  | **Arkray** | *** | | *** | | |  | |  | | |  |  |  | |
|  | **Eurocell** | *** | | *** | | | ** | | * | | |  |  | * | |
|  | **Fortress** | *** | | *** | | |  | | * | | | *** |  | ** | |
|  | **cELISA** | ** | | *** | | | *** | | *** | | | *** | *** |  | |
|  |  |  |  | |  |  | |  | |  |  | | | |  |
|  |  |  |  | |  |  | | Sensitivity | |  | Specificity | | | |  |

**S5. Index and reference test results for each sample.** RBT - the Rose Bengal test (both RBT 1:2 and RBT 1:8 protocols); cELISA - competitive enzyme-linked immunosorbent assay.

**
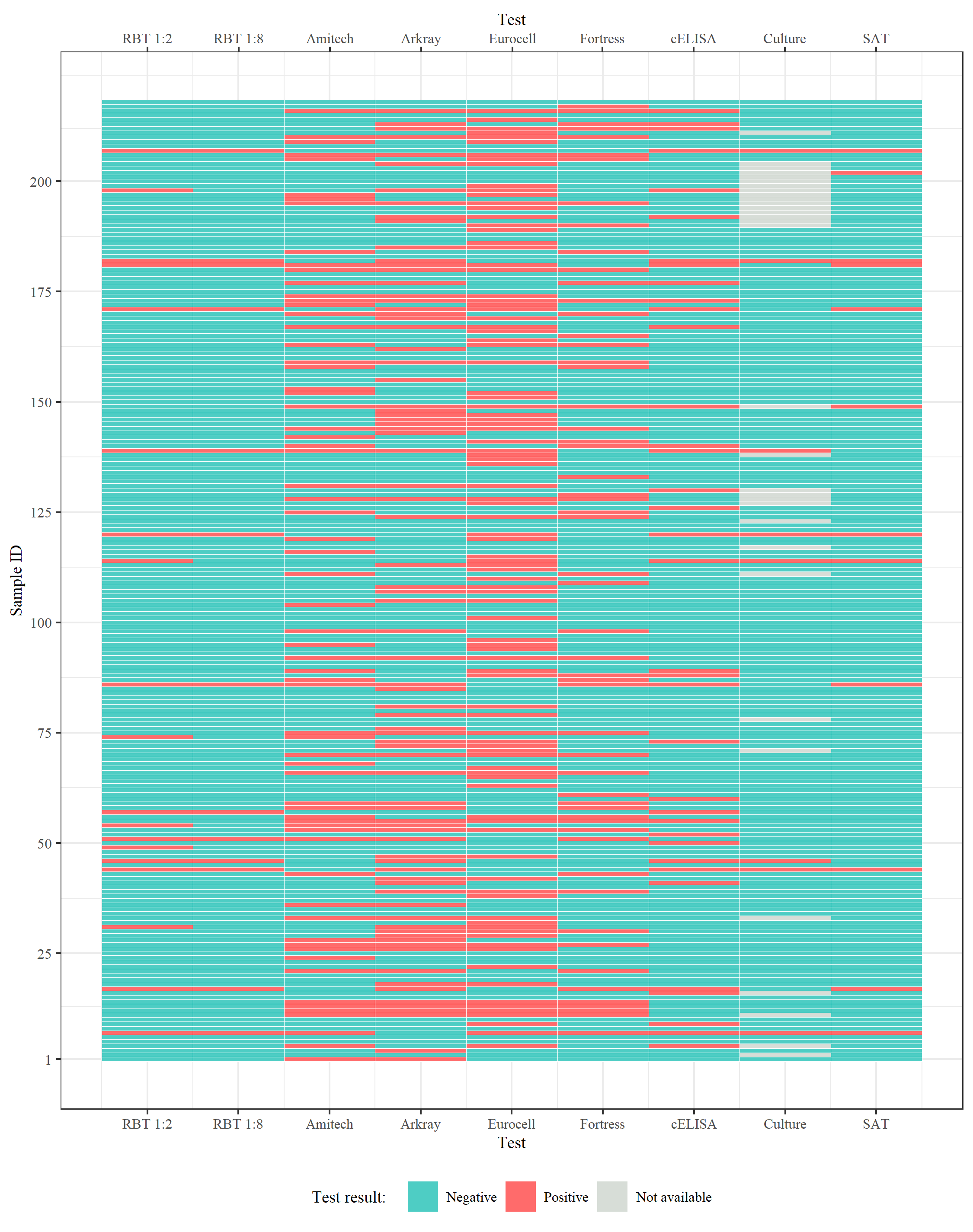
**
